# NAFLD and AATD Are Two Diseases with Unbalanced Lipid Metabolism: Similarities and Differences

**DOI:** 10.3390/biomedicines11071961

**Published:** 2023-07-12

**Authors:** Sara Perez-Luz, Nerea Matamala, Gema Gomez-Mariano, Sabina Janciauskiene, Beatriz Martínez-Delgado

**Affiliations:** 1 Molecular Genetics Unit, Institute of Rare Diseases Research (IIER), Institute of Health Carlos III (ISCIII), 28220 Madrid, Spain; sara.perez@isciii.es (S.P.-L.); nmatamala@isciii.es (N.M.); ggomezm@isciii.es (G.G.-M.); 2 Department of Respiratory Medicine and Infectious Diseases, Biomedical Research in Endstage and Obstructive Lung Disease Hannover BREATH, Member of the German Center for Lung Research DZL, Hannover Medical School, 30625 Hannover, Germany; janciauskiene.sabina@mh-hannover.de; 3 CIBER de Enfermedades Raras, Instituto de Salud Carlos III, CIBERER U758, 28029 Madrid, Spain

**Keywords:** NAFLD, AATD, cardiovascular disease, lipids, meta-inflammation

## Abstract

Non-alcoholic fatty liver disease (NAFLD) is a type of steatosis commonly associated with obesity, dyslipidemia, hypertension, and diabetes. Other diseases such as inherited alpha-1 antitrypsin deficiency (AATD) have also been related to the development of liver steatosis. The primary reasons leading to hepatic lipid deposits can be genetic and epigenetic, and the outcomes range from benign steatosis to liver failure, as well as to extrahepatic diseases. Progressive hepatocellular damage and dysregulated systemic immune responses can affect extrahepatic organs, specifically the heart and lungs. In this review, we discuss the similarities and differences between the molecular pathways of NAFLD and AATD, and the putative value of hepatic organoids as novel models to investigate the physio pathological mechanisms of liver steatosis.

## 1. Non-Alcoholic Fatty Liver Disease (NAFLD)

Non-alcoholic fatty liver disease (NAFLD) is a common liver condition characterized by an excess of lipid accumulation in hepatocytes (steatosis), which is present in about 25% of the adult population [1]. This term includes a range of liver diseases from benign steatosis to cirrhosis, passing through steatohepatitis (NASH) to hepatocellular carcinoma (HCC) [2]. There are different environmental or genetic risk factors that can lead to NAFLD [3], including insulin resistance and obesity. MAFLD (metabolic associated fatty liver disease) has been proposed as a new name that is expected to better mirror the heterogeneities and similarities between NAFLD and metabolic syndrome [4,5]; however, some controversies remain regarding this new name [6].

The pathology typically begins with an altered lipid homeostasis, the intracellular increment of fats followed by an uncontrolled inflammatory response, which can eventually lead to cirrhosis and/or to HCC [7]. Initially, most of the NAFLD patients are asymptomatic and blood markers typically do not reflect liver impairment [8]. The progression to NASH is associated with liver inflammation usually followed by fibrosis, whereas in some cases, the development of liver failure requires liver transplantation. However, cardiovascular diseases (CVD) are among the main causes of death among NAFLD patients [9].

It is widely accepted that free fatty acids act as primary triggers of NAFLD, although there are other factors implicated in disease progression such as dietary habits, obesity, insulin resistance, intestinal microbiota, or epigenetic factors [10]. Patients with NASH typically have high levels of blood endotoxins, suggesting that bacterial endotoxins play a role in NASH pathogenesis [11,12]. Among the intestinal microflora, Gram-negative bacilli seem to be the largest source of endotoxins, such as lipopolysaccharides (LPS). If intestinal enterobacteria invade the portal vein, they inflame the hepatic vasculature leading to persistent inflammation and progressive liver damage. In obese individuals, an increased expression of CD14, an endotoxin co-receptor in the liver, may result in leptin-induced endotoxin hyper responsiveness [13].

Steatosis is defined by the presence of lipid droplets (LDs) in the cytosol of more than 5% of hepatocytes, which is a consequence of altered lipid metabolism when fatty acid obtention exceeds fatty acid removal [14]. Lipid droplets are dynamic organelles composed of neutral lipids, mainly triglycerides and cholesterol esters [15], which act as energy storage but also as protectors against the deleterious effects of free fatty acids [16]. LDs are increasingly recognized as having important non-pathological roles in cell signalling and function. The properties of LDs are highly regulated by proteins coating the surface of LDs to control lipid trafficking and flux [17]. LDs also play roles in endoplasmic reticulum (ER) stress response, protein storage and degradation, and in infection and immunity [18]. Hence, although LDs formation, per se, is not a deleterious event, the accumulation of intrahepatic lipids is associated with increased circulating lipoproteins and increased risk of CVD [19], the main cause of death in NAFLD patients, as mentioned above.

## 2. Alpha-1 Antitrypsin Deficiency (AATD)

Inherited alpha-1 antitrypsin deficiency (AATD) is a rare monogenic disorder (ORPHA 60) mainly related to lung and/or liver diseases, but also to neutrophilic panniculitis or systemic vasculitis [20]. AATD is characterized by low levels of circulating alpha-1 antitrypsin (AAT), an acute phase glycoprotein encoded by the *SERPINA1* gene, in which more than 120 allelic variants have been described [21]. Some mutations in the *SERPINA1* gene have no clinical relevance and are considered as normal variants or M alleles; however, deficient alleles, typically resulting from point mutations or small deletions, are related to low levels or functional activity of AAT, and mild to severe clinical manifestations. Among the deficient alleles, the most clinically relevant and best recognized is the Z allele (Glu342Lys), originating from a point mutation in exon 5 [22]. According to current data, the homozygosity in the Z allele is present in about 96% of AATD patients, whereas the remaining 4% are heterozygous carriers or contain other rare alleles [23].

AAT is primarily synthetized by hepatocytes (about 80%) and acts not only as a main inhibitor of neutrophil elastase and proteinase-3 [24,25], but also as a modulator of caspase activity and apoptosis, as an antioxidant, and/or as a broad immunomodulatory protein [26,27]. The complex tertiary structure of AAT makes it extremely vulnerable to conformational changes, as it happens in the Z allele where a change in just one amino acid triggers AAT polymerization. As mentioned above, AATD mainly affects the liver and lungs; hepatic manifestations are due to AAT intrahepatic polymer accumulation and cytotoxicity [28], whereas lung pathologies are due to low circulating levels, mostly polymeric forms of AAT resulting in an insufficient inhibition of neutrophil proteases [29]. On the other hand, among AATD carriers there is a great variability in clinical presentations: from asymptomatic to those who develop early onset emphysema [30] and/or liver steatosis, fibrosis, cirrhosis, or hepatocarcinoma [31]. This suggests that, in addition to the mutations in *SERPINA1* gene, other genetic and/or environmental factors contribute to the clinical manifestations.

It has been demonstrated that AAT polymers accumulate in the ER by mechanisms that are not completely understood. Although polymer formation triggers the unfolded protein response [32] to be cleared out of the cell by autophagy or the ER-associated degradation (ERAD) pathways, aggregates can remain in hepatocytes, eliciting cellular stress and inflammation, which lead to liver damage [33,34,35].

The liver disease in AATD people with a homozygous Z allele has been associated with liver steatosis [36]. Concordantly, transgenic mice expressing the human Z allele displayed an altered lipid metabolism with increased levels of hepatic triglycerides and cholesterol [37], as well as high numbers of LDs [36]. Likewise, AATD patients with a homozygous Z allele seem to have lower serum levels of cholesterol and triglycerides than non-AATD patients [36], pointing to hampered lipoprotein secretion and a lower risk of CVD [38].

## 3. Meta-Inflammation in NAFLD and AATD

Meta-inflammation is defined as a low-grade chronic inflammation associated with metabolic syndrome [39]. Most scientists agree that meta-inflammation, as a component of immune system, links chronic inflammatory diseases and obesity [40]. In this scenario, adipose tissue macrophages can react to high concentrations of fatty acids and initiate signalling pathways promoting monocyte mobilization and differentiation into macrophages, which further contribute to the inflammatory response [41,42].

**Figure 1:**
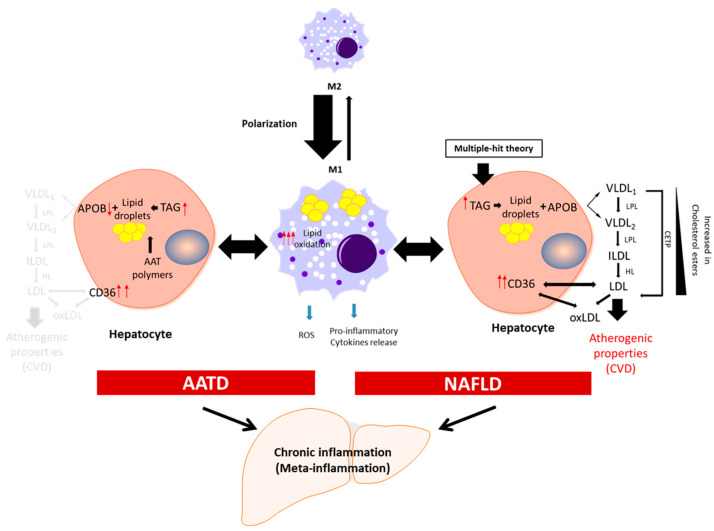
Schematic presentation of the development of chronic inflammation in NAFLD and AATD. Activated liver macrophages promote inflammation characterized by cytokine and free radical (ROS) production and increased lipid oxidation. In this scenario, to diminish the net increment of lipids, hepatocytes fuse triglycerides (TAG) stored in lipid droplets into APOB-containing lipoproteins and increase the expression of the CD36 receptor to export the lipids out of the cells. In NAFLD patients, this increases the plasma lipoprotein levels with the concomitant risk of cardiovascular disease (CVD). In AATD patients, despite the increased expression of CD36, the accumulation of Z-AAT protein impairs lipids secretion and lowers the risk of CVD. LPL: lipoprotein lipase; HL: hepatic lipase; CETP: cholesteryl ester transfer protein; oxLDL: oxidized LDL.

Macrophages derived from hematopoietic progenitors are involved in homeostatic and pathogenic processes. In adult tissues, the functions of macrophages are dependent on the microenvironment, and thus macrophages can acquire a proinflammatory (M1) or an anti-inflammatory (anti-fibrotic) (M2) phenotype [43,44]. Bone-marrow monocyte-derived macrophages can also acquire a pro-inflammatory phenotype and contribute to inflammation [45]. Because of lipid accumulation in NAFLD, not only is macrophage polarization altered in favour of the M1 phenotype, but macrophages also undergo metabolic reprogramming leading to increased fatty acid intake and worsen steatosis [46]. Activated liver Kupffer cells release pro-inflammatory cytokines, which in turn activate hepatic stellate cells, hepatocytes, or endothelial cells [47,48], promoting monocyte infiltration and boosting macrophage population. Furthermore, fat accumulation in Kupffer cells leads to oxidative stress and structural changes in the plasmatic and mitochondrial membranes, while in the context of AATD, due to AAT protein accumulation in the ER, this also leads to activation of the unfolded protein response [14]. An increase in free fatty acids intensifies lipid oxidation, mainly in the mitochondria and peroxisomes, as well as free-radical production, which can lead to mitochondrial damage and fragmentation [49,50]. On the other hand, ER stress induced by misfolded proteins triggering the unfolded protein response elicits p53 expression, mitochondrial cytochrome c release, and apoptosis [51]. Hence, liver Kupffer cells can contribute not only to the sustained meta-inflammation, but also to the progression of NAFLD (Figure 1).

In this scenario, a member of the class B scavenger receptor, CD36, plays a central role. CD36 binds oxidized low-density lipoproteins, long-chain fatty acids, phospholipids, and collagen [52,53]. Its high expression on macrophages, adipocytes, cardiomyocytes, and hepatic cells is important for fatty acid uptake and lipid metabolism. In fact, CD36 expression is much lower in normal hepatocytes than in hepatic steatosis and NAFLD [54]. An increased hepatic CD36 expression can enhance fatty acid uptake and triglyceride accumulation, although the precise role of CD36 in the pathogenesis of fatty liver remains unclear.

In addition to hepatocytes, monocytes and macrophages also express the AAT protein [55,56,57]. A study based on transgenic Z-AAT mice, reproducing most of the liver characteristics of AATD, showed high numbers of liver macrophages [58]. The characterization of these AATD-related macrophages revealed an altered immunophenotype with a population expressing F4/80^hi^ and TIM4neg, known as a contributor in NAFLD progression [59]. As mentioned above, AAT possesses a broad spectrum of anti-inflammatory properties [60], whereas polymers of Z-AAT are pro-inflammatory, and their accumulation in monocytes and macrophages may trigger NLRP3 inflammasome activation [61]. Kupffer cells also express and accumulate the Z-AAT protein [62], but the effect of Z-AAT accumulation on AATD progression remains to be clarified [59].

## 4. Features of Lipid Metabolism in NAFLD and AATD

Lipids are key cellular components involved in maintaining the integrity of cellular membranes and energy homeostasis, although they also contribute to pathologies [63]. Lipid homeostasis in the liver depends on the equilibrated balance between lipid acquisition (de novo formation and uptake), storage, and removal [64]. Neutral lipids (sterol esters and triglycerides) are stored in LDs, and in a healthy liver, these lipids do not exceed 5% [65]. Fatty acids stored as sterol esters and triglycerides are used during liver homeostasis to generate energy via fatty acid oxidation or are transported to other organs in very-low-density lipoprotein (VLDL) [66,67] (Figure 1).

A composite route required for VLDL assembly is the lipidation of APOB100, a main and highly hydrophobic apolipoprotein. Initially, VLDLs are pre-assembled in ER lumen by the microsomal triglyceride transfer protein [68] and are subsequently moved to the secretory pathway as VLDL2 particles (TAG poor). These particles are secreted out of the hepatocytes or undergo additional lipidation through LD fusion and become VLDL1 particles (TAG enriched) [69]. A failure in APOB lipidation triggers its degradation because the protein is unable to fold correctly [70]. Therefore, VLDL secretion regulates the fat amount in the liver, and VLDL production and secretion are considered as contributors to CVDs [71]. An imbalanced lipid metabolism in NAFLD patients is related to higher levels of VLDL production (and consequently VLDL secretion), which links NAFLD with CVDs.

NAFLD is a multifactorial disorder, in which genetic alterations play a role [72]. For example, genes such as *PNPLA3* [73] and *TM6SF2* [74] are linked to a high risk of NAFLD. The patatin-like phospholipase domain-containing 3 gene (*PNPLA3*) encodes a membrane-associated lipase that mediates triacylglycerol hydrolysis to manage the increasing amount of lipids after a meal intake. The nonsynonymous transversion from cytosine to guanine (rs738409) renders an amino acid change at codon 148 (isoleucine to methionine) that results in an imbalance between the liver triglyceride content and VLDL secretion [75]. The results found by the authors point to a reduced mobilization of triacylglycerols from lipid droplets, even though VLDL assembly itself is not damaged or diminished, which could justify why this variant is not associated with a risk of CVD [75].

The transmembrane 6 superfamily member 2 (*TM6SF2*) gene, mainly expressed by hepatocytes, enterocytes, and renal cells, encodes for a protein located either in the ER membrane or in the ER−Golgi intermediate compartment. This protein participates in triglyceride secretion and LD formation, and thus regulates the liver fat content. A variant of the *TM6SF2* gene (glutamic acid 167 to lysine) is implicated in reduced VLDL secretion [74]. Despite contradictory results in a mouse model using protein overexpression or silencing, lipid accumulation in humans has proven that this variant of the *TM6SF2* gene is responsible of the reduced hepatic secretion of VLDL1, which is generated by the combination of VLDL2 and LDs. One putative explanation for this finding is the inability of the variant protein to stabilize APOB [76]. Hence, similarly to the above-described variant of the *PNPLA3* gene, despite fat accumulation in the liver, there is no additional risk for CVD. These latter gene variants, to some extent, resemble the situation observed in AATD patients with liver disease, in which diminished hepatic lipid secretion and reduced risk of ischemic heart disease have been reported [36,38].

The lipid components of low-density lipoproteins (LDL) are involved in oxidation reactions, generating a variety of oxidized-lipid-derived products found in atheroma plaques [77]. The intracellular uptake of these oxidized products is mediated by the CD36 [78,79,80], a fatty acid translocase and signalling molecule acting as a receptor for lipoproteins; its derivatives [81]; and free fatty acids [82]. The expression of CD36 is upregulated in NAFLD [83,84,85]. Some studies based on mice models suggest that CD36 is a negative regulator of the autophagy and lipophagy induction. In line with this, *CD36* knockdown in HepG2 cells increases lipophagy and β-oxidation, which contribute to lipid accumulation [86].

An increase in *CD36* expression has also been linked with AATD [87]. Moreover, a functional relationship between AAT and CD36 has been described, where AAT prevented inflammasome activation in monocytes/macrophages through a signalling cascade involving CD36 [88].

## 5. Relationships between NAFLD, AATD, and Chronic Obstructive Pulmonary Disease (COPD)

NAFLD is a progressive liver disease evolving via NASH and fibrosis to cirrhosis, and eventually to hepatocellular carcinoma [89]. Investigations demonstrate that NAFLD, NASH, and liver fibrosis are prevalent in patients with COPD (by 41%, 37%, and 61%, respectively) [90]. Patients with COPD and NASH seem to have elevated TNF-α and leptin levels, unlike patients with COPD without liver damage [91]. It is thought that the chronic inflammatory synergy between NAFLD/NASH and COPD can trigger further injury and the progression of both diseases [91,92,93]. Additional studies are warranted to answer questions whether NAFLD and COPD develop together or separately.

AATD is the most common genetic cause of emphysema, and, as a result, the lack of normal levels of AAT do not protect the lungs from damage, leading to an increased risk for developing COPD. Subjects with homozygous ZZ and heterozygous MZ AATD genotypes seem to also have a higher risk of developing NAFLD than non-deficient subjects [94]. For decades, intravenous therapy with human-plasma-purified AAT has been used to treat patients with AATD-related emphysema. The lung-protective effects of AAT are attributed to the inhibition of proteases involved in lung matrix fragmentation, macrophage activation, and endothelial cell apoptosis [95]. Therapy with AAT can rescue epithelial cells from free-heme-mediated pro-inflammatory activation, cell death, and dysfunctional autophagy [96], and prevent acute lung rejection in a mouse orthotopic lung transplantation model [97]. We have also demonstrated hepatoprotective effects of AAT in three different mouse models of acute liver failure (ALF) via the inhibition of caspase-3 and TNF-α, which was also confirmed in a model of alcoholic steatohepatitis [98,99]. Moreover, AAT has been reported to inhibit the process of renal fibrosis through the suppression of TGF-β/Smad3 signalling [100], and to lower the liver stiffness, liver fibrosis, and lower levels of liver enzymes [36]. In INS-1E cells or a primary rat pancreatic islet model, we previously demonstrated that AAT increases insulin secretion in a glucose-dependent manner and protects INS-1E cells from cytokine-induced apoptosis [101]. Patients with NAFLD are associated with hepatic and adipose tissue insulin resistance, and typically have higher levels of serum TNF-α and IL-6, soluble TNFR1 (sTNFR1), and soluble IL-6 receptor (sIL-6R) than patients with a simple steatosis without signs of inflammation, ballooning cells, or fibrosis [102]. Persistent inflammation and altered lipid homeostasis may lead to the evolution of both NAFLD and COPD. In this context, therapy with the AAT protein is of great interest because of its broad tissue-protective effects, which might be beneficial for both NAFLD and COPD patients.

## 6. Organoids to Model Liver Disease

In vitro two-dimensional (2D) cell models are widely used to reproduce the physiopathology and molecular mechanisms of various diseases. Traditionally used human cell lines are relatively cheap, easy to handle, and can be genetically modified. Nevertheless, the use of cell lines to address questions related to specific human diseases is not always straightforward, and they also lack tissue organization and grow without a physiological context. Therefore, the more recently developed three-dimensional (3D) cell cultures have become better experimental tools [103]. The 3D cultures known as organoids can be generated from adult stem cells, embryonic stem cells (ESCs), or induced pluripotent stem cells (iPSCs). Organoids derived from human progenitor cells can be long-term expanded; they can recapitulate organ architecture with remarkable fidelity, with the presence of multiple cell types of the specific organ [104,105]; and they assume at least some functions of the organ. All these reasons make them a valuable tool for testing new therapeutic drugs and for disease modelling to investigate human diseases including neural disorders [106,107], cancer [108,109], lung diseases [110,111,112], liver diseases [113,114], and others.

In 2001, for the first time, Michalopoulos and colleagues described 3D liver organoids derived from rat hepatocytes [115] that had short-term survival in the culture. A long-term maintenance of cultured organoids was achieved in 2013 by Huch and colleagues from adult murine tissue by using matrigel, hepatocyte growth factor (HGF), epidermal growth factor (EGF), and factors induced under liver damage such as fibroblast growth factor and R-spondin [116]. In parallel, Takebe and collaborators described an alternative method to obtain liver organoids by combining human iPSC-derived hepatocytes, mesenchymal stem cells, and umbilical cord cells [117]. Subsequently, these modified protocols became useful for the modelling of different diseases [118]. Furthermore, patient-derived organoids are an excellent tool to study personalized presentations of pathophysiological conditions influenced by genetic and/or environmental factors.

Hepatic organoids generated from AATD patients have been proven as a new tool to study the pathophysiological characteristics of the liver [119,120]. These organoids have typical intrahepatic retention of Z-AAT polymers and show a positive diastase-resistant (PAS) staining [119,120]. Additionally, when AATD hepatic organoids were differentiated into hepatocytes, Huch and collaborators showed that these cells presented high ER stress and apoptosis [120]. Gomez-Mariano and colleagues further confirmed that differentiated liver organoids express albumin (*ALB*) and apolipoprotein B (*APOB*) genes, two specific hepatocyte markers [119].

Hepatocytes or hepatic parenchymal cells comprise approximately 60% of all liver cells, which form a 3D lattice filled by hepatic sinusoids. This latter provides nourishment for the parenchymal cells of the 3D structure, inter-luminal Kupffer, sinusoidal endothelial cells, and perisinusoidal stellate cells [121]. Different 3D cell strategies have been used for NAFLD modelling because the progression of this disease depends on the interactions among multiple liver cell types [122,123]. For example, Shen and collaborators treated hepatic stellate cells (HSC) with conditioned media from patient-derived liver organoids and proved that hepatocyte-derived VEGFA induces HSC activation and hepatocarcinoma progression, even in the absence of increased lipid accumulation [124]. Another NAFLD 3D model in vitro was based on the co-cultures of HepG2 (hepatocyte cell line) and LX-2 (HSC), forming hepatic spheroids and accumulating intracellular lipids after exposure to free fatty acid (FFA) [125]. To reproduce the NASH condition, spheroids were also generated using co-cultures of hepatocytes, HSC, and macrophages (ratio 4:1:1) in a culture medium containing high glucose and palmitate. In this model, treatment with the anti-CD47 antibody, a new therapeutic in obesity, did not improve steatosis, but reduced fibrosis and liver inflammation by inhibiting neutrophil and macrophage activation [126]. Likewise, the combination of hepatocytes (80%), Kupffer cells (10%), HSC (5%), and endothelial cells (5%) have been used to test a protective role of miR-122 in NAFLD. It has been suggested that miR-122 affects steatosis, fibrosis, and altered lipid metabolism because it changes the expression of lipases and fatty acid binding proteins implicated in intrahepatic lipid accumulation [127].

Other approaches to generate liver organoids are based on the differentiation of iPSCs [128]. To reproduce NAFLD, iPSCs-derived liver organoids were exposed to different free fatty acid treatments and analysed for the enlargement of hepatocytes (ballooning), as well as for organoids stiffness by atomic force microscopy [128]. More sophisticated approaches were also applied to deepen our knowledge regarding NAFLD development. For example, sirtuin-1 has been implicated in the progression of NAFLD due to its effects on de novo lipogenesis and beta-oxidation [129,130]. Different types of 3D models for NAFLD have been reviewed by Park and colleagues [131] and by Wang and collaborators [122].

Liver organoids generated from AATD patient-related liver disease seem to recapitulate hepatocyte alterations [119]. Like mature hepatocytes, differentiated hepatic organoids express albumin and apolipoprotein B, although it was found that Z-AATD-derived organoids show a lower expression of both genes, *ALB* and *APOB* [119]. A transcriptomic analysis carried out by our group confirmed the reduced APOB expression in Z-AATD organoids, which could at least partially explain why ZZ patients, despite their increased level of hepatic steatosis, show reduced levels of serum triglycerides and VLDL lipoproteins [36]. In addition to this downregulation of *APOB* transcription levels, other factors may contribute to the final amount of APOB [132]. Among differentially expressed genes, we also found *CD36* as one of the upregulated genes in Z-AATD organoids when compared with the controls, somehow mimicking the behaviour displayed in NAFLD. The AATD-related steatosis is likely favoured by the increased expression in the CD36 receptor, although some work is still needed to unravel the relationship between them. On the other hand, the reduced APOB protein (Figure 1) might contribute to the diminished circulating levels of lipoproteins [36,133] and reduced risk of CVDs [38]. Yet, the association between AATD and CVDs requires additional studies.

## 7. Conclusions

NAFLD and AATD are hepatic diseases characterized by increased hepatic lipid content and consequently the intracellular accumulation of lipid droplets. To maintain liver homeostasis, hepatocytes in NAFLD eliminate the excess of lipids by exporting them to the bloodstream as lipoproteins, which results in an increased risk of cardiovascular disease in NAFLD patients. Conversely, in patients with AATD, the intrahepatic accumulation of misfolded AAT protein lowers lipid secretion and thus risk for cardiovascular disease. The use of patient-derived liver organoids as new cellular models is of great value, especially for the development of new personalized therapies, as well as for studying the underlying molecular mechanisms in NAFLD and AATD.

## Data Availability

This study did not report any data.
